# Associations between breath acetone overnight dynamics and obstructive sleep apnea manifestations

**DOI:** 10.1186/s12890-025-04021-0

**Published:** 2025-11-27

**Authors:** Yen-Hao Su, I-Jung Liu, Wen-Te Liu, Rachel Chien, Ying-Ying Chen, Yen-Ling Chen, Arnab Majumdar, Jiunn-Horng Kang, Kang-Yun Lee, Po-Hao Feng, Kuan-Yuan Chen, Yi-Chun Kuan, Hsin-Chien Lee, Chien-Ling Su, Cheng-Yu Tsai

**Affiliations:** 1 Division of General Surgery, Department of Surgery, Taipei Medical University- Shuang Ho Hospital, New Taipei City, 23561 Taiwan; 2 Department of Surgery, School of Medicine, College of Medicine, Taipei Medical University, Taipei, 11031 Taiwan; 3 TMU Research Center of Cancer Translational Medicine, Taipei Medical University, Taipei, 11031 Taiwan; 4 Metabolic and Weight Management Center, Taipei Medical University-Shuang Ho Hospital, New Taipei City, 23561 Taiwan; 5 Research Center of Sleep Medicine, College of Medicine, Taipei Medical University, Taipei, 11031 Taiwan; 6 School of Respiratory Therapy, College of Medicine, Taipei Medical University, Taipei, 11031 Taiwan; 7 Division of Pulmonary Medicine, Department of Internal Medicine, Taipei Medical University-Shuang Ho Hospital, New Taipei City, 23561 Taiwan; 8 Advanced Technology Lab, Wistron Corporation, Taipei, 11469 Taiwan; 9 Sleep Center, Taipei Medical University-Shuang Ho Hospital, New Taipei City, 23561 Taiwan; 10 Research Center of Artificial Intelligence in Medicine, Taipei Medical University, Taipei, 11031 Taiwan; 11 Center for Artificial Intelligence and Advanced Robotics, National Taiwan University, Taipei, 106319 Taiwan; 12 College of Biomedical Engineering, Taipei Medical University, Taipei, 11031 Taiwan; 13 Department of Civil and Environmental Engineering, Imperial College London, London, SW7 2AZ UK; 14 Department of Physical Medicine and Rehabilitation, Taipei Medical University Hospital, Taipei, 11031 Taiwan; 15 Graduate Institute of Nanomedicine and Medical Engineering, College of Biomedical Engineering, Taipei Medical University, Taipei, 11031 Taiwan; 16 Department of Neurology, Taipei Medical University-Shuang Ho Hospital, New Taipei City, 23561 Taiwan; 17 Department of Neurology, School of Medicine, College of Medicine, Taipei Medical University, Taipei, 11031 Taiwan; 18 Taipei Neuroscience Institute, Taipei Medical University, Taipei, 11031 Taiwan; 19 Department of Psychiatry, Taipei Medical University Hospital, Taipei, 11031 Taiwan; 20 TMU Research Center of Biomedical Devices, Taipei Medical University, 250 Wuxing Street, Taipei, 11031 Taiwan; 21 School of Biomedical Engineering, College of Biomedical Engineering, Taipei Medical University, 250 Wuxing Street, Taipei, 11031 Taiwan; 22 TMU Research Center for Thoracic Medicine, Taipei Medical University, Taipei, 11031 Taiwan

**Keywords:** Obstructive sleep apnea (OSA), Breath acetone, Apnea-hypopnea index (AHI), Arousal index (ArI), Polysomnography (PSG)

## Abstract

**Objective:**

Obstructive sleep apnea (OSA) is associated with metabolic dysregulation, potentially affecting ketone body metabolism. However, relationships between OSA and ketone body metabolism require further investigation. Thus, in this study, we investigated relationships between breath acetone levels and OSA manifestations (e.g., hypoxia and arousal events).

**Methods:**

Baseline characteristics were collected from 66 eligible participants, and breath acetone levels were measured before and after polysomnography (PSG). Participants were categorized into two groups using the apnea-hypopnea index (AHI): normal-to-mild (< 15 events/h) and moderate-to-severe (≥ 15 events/h) OSA groups. Differences in baseline characteristics, PSG parameters, and breath acetone levels between the two groups were analyzed. Next, correlations analyses and multivariable regression models adjusted for baseline characteristics were performed to examine relationships between alterations in breath acetone levels and various sleep parameters.

**Results:**

Significant differences were observed between the two groups in baseline characteristics, sleep quality indices and the overnight difference in acetone levels (all *p* < 0.01). Additionally, the overnight difference in acetone levels exhibited significant negative correlations (*p* < 0.01) with the AHI, arousal index (ArI), and snoring index. In regression models adjusted for confounding factors, this overnight acetone difference remained significantly and negatively associated with both AHI and ArI values (both *p* < 0.05).

**Conclusions:**

The present observations provide insight into potential links between overnight breath acetone alterations and OSA manifestations.

**Supplementary Information:**

The online version contains supplementary material available at 10.1186/s12890-025-04021-0.

## Brief summary

In this study, we investigated relationships between obstructive sleep apnea (OSA) manifestations and breath acetone levels. Baseline characteristics and polysomnography (PSG) data were obtained from 66 eligible participants, with breath acetone levels measured before and after sleep to calculate overnight differences. Multivariable regression models, adjusted for baseline characteristics, revealed significant negative associations between the overnight difference in breath acetone levels and both the apnea-hypopnea index (AHI) and arousal index (ArI). These findings suggest potential metabolic links between breath acetone alterations and OSA-related hypoxia and arousal events.

## Introduction

Obstructive sleep apnea (OSA) is an emerging global health concern characterized by recurrent upper airway collapse, leading to intermittent hypoxia and frequent arousal [1, 2]. An estimated 26% of individuals aged 30–70 years have OSA, with around 10% exhibiting an apnea-hypopnea index (AHI) of ≥ 15 events/h [3]. OSA is linked to chronic illnesses, including cardiovascular diseases, cognitive impairment, and hypertension [4, 5]. Furthermore, it contributes to insulin resistance, obesity, metabolic disorders, and an increased risk of mortality [6, 7].

OSA is associated with metabolic dysregulation, potentially affecting both the production and utilization of ketone bodies. Ketone bodies serve as an alternative energy source to glucose and are key byproducts of lipid metabolism [8]. Their generation and usage are closely aligned with circadian rhythms [9], helping synchronize cellular metabolism with the sleep-wake and feeding-fasting cycles [10, 11]. In OSA, repeated hypoxia-reoxygenation cycles contribute to metabolic disturbances and systemic health complications [12, 13]. Notably, intermittent hypoxia was shown to influence ketogenesis and β-oxidation by modulating levels of non-esterified fatty acids [14, 15]. A breath acetone analysis has garnered attention as a noninvasive and accessible approach for monitoring ketogenesis [16, 17]. Additionally, studies reported a strong correlation between breath acetone and blood ketone concentrations, underscoring its potential as a reliable ketogenesis indicator [18]. A previous study reported lower breath acetone concentrations in OSA patients compared to healthy controls [19]. However, another study found no significant overnight changes in breath acetone levels among 10 OSA patients [20]. These findings illustrate an unclear link between OSA-induced intermittent hypoxia and acetone fluctuations, necessitating further elucidation.

Recent studies suggested that ketone bodies enhance sleep quality by increasing rapid-eye-movement (REM) sleep and reducing wakefulness after sleep onset (WASO) [21, 22]. Frequent sleep arousals drive sympathetic surges, leading to metabolic alterations, including ketogenesis [23, 24]. One study reported elevated daytime breath acetone levels in individuals with arousal-induced sleep fragmentation compared to healthy controls [25]. In bidirectional relationships, ketone bodies can suppress sympathetic activity under ketogenic conditions to maintain homeostasis [26], while also modulating neurotransmitters to reduce neuronal excitability and regulate sleep patterns [27, 28]. Specifically, acetone exerts an anticonvulsant effect [29], contributing to neural stability and oxidative stress mitigation [30]. However, another study found no significant correlation between breath acetone fluctuations and spontaneous arousals or awakenings in healthy subjects [31]. Therefore, the association between variations in breath acetone levels and sleep arousal remains uncertain and warrants further investigation.

In this study, we explored relationships between OSA manifestations (e.g., hypoxia and arousal events) and breath acetone levels. We collected baseline characteristics and measured breath acetone concentrations before and after polysomnography (PSG). These data were compared between two groups: normal-to-mild OSA and moderate-to-severe OSA. Next, correlations and associations between alterations in breath acetone levels and various sleep parameters were examined. The present findings may provide insights into a potential link between OSA severity and metabolic alterations, as indicated by variations in breath acetone levels.

## Methods

### Study design and population

**Fig. 1:**
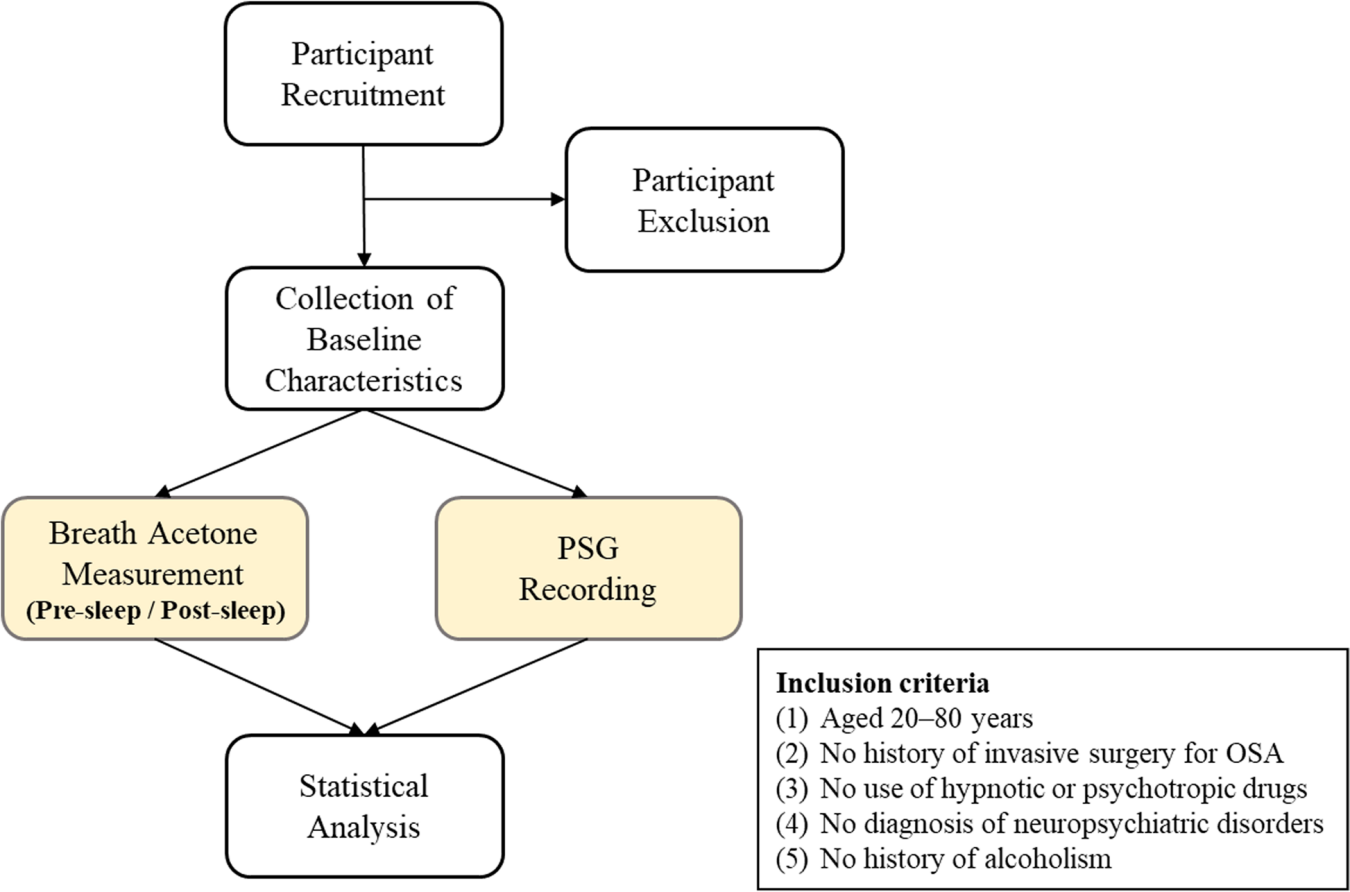
Study protocol. Participants were recruited from the Sleep Center of Taipei Medical University–Shuang Ho Hospital according to predefined inclusion criteria. Baseline characteristic were recorded before undergoing PSG. During PSG, breath acetone levels were measured using a portable device before sleep and after waking. All collected data were subsequently analyzed. Abbreviations: OSA, obstructive sleep apnea; PSG, polysomnography

This study enrolled patients suspected of having OSA and who were referred to the Sleep Center of Taipei Medical University–Shuang Ho Hospital (New Taipei City, Taiwan). Inclusion criteria for participants were: (1) aged 20–80 years, (2) a PSG recording duration of > 6 h with the occurrence of REM stage sleep, (3) no previous invasive surgery for OSA, (4) no use of hypnotic or psychotropic drugs, (5) no diagnosis of neuropsychiatric disorders (e.g., neurodegenerative diseases, epilepsy, stroke, schizophrenia, or bipolar disorder), and (6) no receipt of a diagnosis of Type 1 diabetes or alcoholism. The study procedure is illustrated in Fig. 1. Baseline characteristics, including age, sex, body-mass index (BMI), neck circumference, and waist circumference, were collected from eligible participants. Data on comorbidities (e.g., Type 2 diabetes) and lifestyle factors (e.g., smoking status and alcohol consumption) were obtained from electronic medical records. To minimize effects from diet and alcohol, the participants were instructed to fast for at least 3 h before the start of PSG until waking the next morning. Breath acetone levels were measured before sleep and after waking. All collected data were used for subsequent analysis.

### PSG

The PSG examination was conducted in a sleep laboratory using a ResMed Embla N7000 (ResMed, San Diego, CA, USA) and an Embla MPR (ResMed Global Supplier Alliance, Sydney, Australia). Recorded signals were analyzed and scored using RemLogic software (vers. 3.41, Embla, Thornton, CO, USA) by licensed technicians in accordance with guidelines published by the American Academy of Sleep Medicine (AASM) in 2017 [32]. To minimize bias, all technicians underwent monthly training, and each scoring outcome was independently reviewed by two technicians. In cases of discrepancies, consensus was reached through discussion. Sleep parameters of interest included sleep architecture (wake, N1, N2, and N3 stages), hypoxia, respiratory events, and arousal. Specifically, sleep quality indices included the AHI, oxygen desaturation index for ≥ 3% (ODI), snoring index (SI), and arousal index (ArI) [33]. Participants were classified into two groups based on the AHI: normal-to-mild (AHI < 15 events/h) and moderate-to-severe (AHI ≥ 15 events/h), in accordance with AASM guidelines [34].

### Breath acetone measurement

A portable breath acetone measurement device (Aceto20, CareExpert Technology, Zhubei City, Taiwan) equipped with tungsten trioxide (WO_3_) sensors, was used to assess breath acetone levels (in ppm). The accuracy, stability, and sensitivity of breath WO_3_-based breath analyzers in detecting acetone levels were previously validated [35, 36]. Upon exposure to acetone, oxygen ions adsorbed onto the WO_3_ sensor surface interact with acetone molecules, resulting in impedance changes. These changes are then processed to determine acetone levels. Standard acetone gas, in the range of 5–20 ppm, was used to calibrate acetone determinations of the devices.

Each measurement followed the standard protocol provided by the manufacturer under the supervision of at least one researcher. First, the device was heated for 1–2 min before each measurement to facilitate absorption of sufficient oxygen ions. Participants were then instructed to take a deep breath to empty their lungs before inhalation. During an acetone measurement, they were required to exhale smoothly for 3–5 s to ensure sufficient airflow for detection. If a participant failed to maintain smooth exhalation or the device failed to produce results, the measurement was repeated. Acetone levels were recorded pre-sleep and post-sleep, and the overnight difference (post-sleep minus pre-sleep values) was calculated. All devices underwent periodic calibration by the manufacturer to maintain consistency and accuracy.

### Statistical analysis

An open-source Python library, Scikit-learn (vers. 0.21.2, Python Software Foundation, Fredericksburg, VA, USA), was used for all statistical analyses [37]. First, the Shapiro-Wilk test was applied to continuous variables to assess the normality of their distribution. For variables with a normal distribution (Shapiro-Wilk test, *p* > 0.05), Student’s *t*-test was performed, whereas the Mann-Whitney U-test was employed for non-normally distributed variables (Shapiro-Wilk test, *p* < 0.05). As to nominal variables, Chi-squared tests were conducted to compare intergroup differences. Additionally, a Spearman correlation analysis was used to determine the correlations between sleep quality indices and breath acetone levels. Multivariable linear regression models were applied to explore the associations between sleep quality indices and the overnight difference in breath acetone levels after adjustment for confounding factors. Regression results are reported as beta coefficients with 95% confidence intervals (CIs). Statistical significance was defined as *p* < 0.05 for all tests.

## Results

### Comparisons of demographics and breath acetone levels

Participants were stratified into two groups: normal-to-mild (*n* = 24) and moderate-to-severe (*n* = 42) groups, with comparisons of baseline characteristics and acetone levels presented in Table 1. The moderate-to-severe group was predominantly male and had a significantly higher mean age compared to the normal-to-mild group (37.75 ± 12.14 vs. 47.71 ± 10.05 years, *p* < 0.01). Additionally, the moderate-to-severe group exhibited significantly higher mean BMI, neck circumference, and waist circumference values than the normal-to-mild group (all *p* < 0.01) did. The prevalence of Type 2 diabetes did not differ significantly between the two groups (3/21 vs. 5/37, *p* = 0.99). Additionally, no significant differences were observed in smoking status or alcohol consumption (both *p* > 0.50). Specifically, alcohol consumption frequency was 1–2 units/week among all participants who consumed alcohol.

**Table 1:** Comparisons of baseline characteristics and breath acetone levels categorized by obstructive sleep apnea severity (*N* = 66)

Variable	Normal-to-mild group (*N* = 24)	Moderate-to-severe group (*N* = 42)	*P* value
Age (years)^a^	37.75 ± 12.14	47.71 ± 10.05	< 0.01
Sex (male/female)^b^	7/17	29/13	< 0.01
BMI (kg/m^2^)^a^	23.65 ± 4.22	27.63 ± 4.82	< 0.01
Neck circumference (cm)^a^	33.62 ± 2.76	37.75 ± 3.96	< 0.01
Waist circumference (cm)^a^	80.50 ± 10.34	93.94 ± 11.83	< 0.01
Acetone level (ppm)^c^			
Presleep	2.30 ± 0.29	2.36 ± 0.45	0.88
Postsleep	2.34 ± 0.44	2.13 ± 0.37	0.07
Overnight Difference	0.04 ± 0.28	−0.23 ± 0.28	< 0.01
Type 2 Diabetes (yes/no)^b^	3/21	5/37	0.99
Alcohol consumption (yes/no)^b^	1/23	3/39	0.99
Smoking status^b^			0.54
Never	21 (87.50%)	32 (76.20%)	
Current	2 (8.30%)	7 (16.70%)	
Quit	1 (4.20%)	3 (7.10%)	

Data are expressed as the mean ± standard deviation or number (percentage). Alcohol consumption: 1–2 units/week

*Abbreviations*: *BMI* Body-mass index

*p* values were derived from the ^a^Student’s *t*-test, ^b^Chi-squared test, or ^c^ Mann-Whitney U-test

Regarding breath acetone levels, the overnight difference was significantly lower in the moderate-to-severe group (−0.23 ± 0.28 ppm) compared to the normal-to-mild group (0.04 ± 0.28 ppm, *p* < 0.01). However, absolute pre-sleep and post-sleep acetone levels did not significantly differ between the two groups. The results of a subgroup analysis of acetone measurements among the participants without diabetes (*n* = 58) is presented in Supplementary Table S1. In this subgroup, the overnight difference in acetone levels also significantly differed between the two groups (− 0.23 ± 0.28 vs. 0.03 ± 0.26 ppm, *p* < 0.01).

### Comparisons of sleep parameters

Table 2 presents comparisons of sleep parameters derived from PSG reports. The moderate-to-severe group exhibited significantly lower sleep efficiency compared to the normal-to-mild group (72.42% ± 8.78% vs. 78.39% ± 7.61%, *p* = 0.01), with a similar trend observed for total sleep time (292.23 ± 32.42 vs. 269.74 ± 33.65 min, *p* = 0.01). Regarding oxygen saturation (SpO_2_) levels, both the mean and minimum SpO_2_ were significantly lower in the moderate-to-severe group than in the normal-to-mild group (both *p* < 0.01). Next, the average minutes of WASO and the percentage of time spent in the wake stage were significantly higher in the moderate-to-severe group compared to the normal-to-mild group (both *p* < 0.05).

**Table 2:** Comparisons of polysomnography parameters categorized by obstructive sleep apnea severity (*N* = 66)

Variable		Normal-to-mild group (*N* = 24)	Moderate-to-severe group (*N* = 42)		*p*
Sleep efficiency (%)^a^	78.39 ± 7.61		72.42 ± 8.78	0.01	
Mean SpO_2_ (%)^a^	95.72 ± 1.36		94.09 ± 1.53	< 0.01	
Minimum SpO_2_ (%)^c^	79.49 ± 30.53		77.04 ± 18.47	< 0.01	
WASO (min)^c^	41.08 ± 23.84		56.52 ± 30.28	0.03	
Total sleep time (min)^a^	292.23 ± 32.42		269.74 ± 33.65	0.01	
Sleep stage (% of SPT)					
Wake^c^	12.33 ± 7.11		17.20 ± 8.67	0.02	
NREM^c^	69.54 ± 19.67		71.13 ± 8.20	0.46	
REM^a^	12.77 ± 6.22		11.65 ± 5.75	0.47	
Sleep quality index (events/h)^c^					
AHI	7.39 ± 4.13		36.96 ± 16.96	< 0.01	
ODI	10.28 ± 24.86		25.06 ± 15.82	< 0.01	
SI	77.73 ± 150.64		266.23 ± 196.71	< 0.01	
ArI	23.81 ± 12.58		35.14 ± 16.72	< 0.01	

Data are expressed as the mean ± standard deviation

*Abbreviations:* *SpO*_2_, Oxygen saturation measured by pulse oximetry, *WASO* Waking after sleep onset, *SPT* Sleep period time, *NREM* Non–rapid eye movement, *REM* Rapid-eye-movement, *AHI* Apnea–hypopnea index, *ODI* Oxygen desaturation index for ≥ 3%, *SI* Snoring index, *ArI* Arousal index

*p* values were derived from the ^a^Student’s t-test, ^b^Chi-squared test, or ^c^Mann-Whitney U-test

Regarding sleep quality indices, the ArI was significantly higher in the moderate-to-severe group than in the normal-to-mild group (35.14 ± 16.72 vs. 23.81 ± 12.58 events/h, *p* < 0.01). Similar differences were observed for the AHI, ODI and SI between the two groups (all *p* < 0.01). Supplementary Table S1 presents the results of a comparison of sleep quality index values among the participants without diabetes. In this subgroup, all indices were significantly higher in the individuals with moderate-to-severe OSA (*n* = 37) than in those with normal-to-mild OSA (*n* = 21).

### Correlations between sleep quality indices and breath acetone levels

**Fig. 2:**
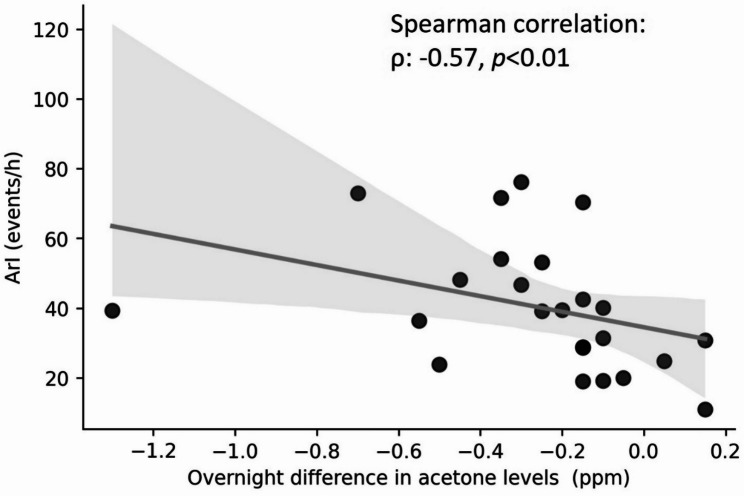
Correlation between the arousal index (ArI) and overnight difference in breath acetone levels among participants with severe obstructive sleep apnea (OSA). In the severe OSA subgroup (apnea-hypopnea index (AHI) ≥ 30 events/h, *n* = 24) of the moderate-to-severe group (*n* = 42), ArI values were significantly and negatively correlated with the overnight difference in breath acetone levels

Correlations between sleep quality indices and breath acetone levels are illustrated in Table 3. The AHI and SI were significantly correlated with the overnight difference in acetone levels (AHI: ρ: −0.45, *p* < 0.01; SI: ρ: −0.28, *p* < 0.05). Likewise, the ArI showed a significant moderate negative correlation with the overnight difference in acetone levels (ρ: −0.45, *p* < 0.01). Fig. 2 presents the significant negative correlation between the ArI and overnight difference in acetone levels among participants with severe OSA (AHI ≥ 30 events/h, *n* = 24; ρ: −0.57, *p* < 0.01).

**Table 3:** Correlations between sleep quality index values and breath acetone levels (*N* = 66)

Variable	Acetone level (ppm)		
	Presleep	Postsleep	Overnight difference
Sleep quality index (events/h)			
AHI	−0.03	−0.29 ^*^	−0.45 ^**^
ODI	−0.06	−0.17	−0.22
SI	−0.09	−0.23	−0.28 ^*^
ArI	0.20	−0.04	−0.45 ^**^

*Data are expressed as coefficients.*

*Abbreviations*: *AHI* Apnea-hypopnea index, *ODI* Oxygen desaturation index for ≥ 3%, *SI* Snoring index, *ArI* Arousal index

^*^p < 0.05

^**^p < 0.01

Similar results were observed among the participants without diabetes (Supplementary Table S2). The overnight difference in breath acetone levels was significantly correlated with the ArI (ρ: −0.438, *p* < 0.01). Negative correlations were observed with the AHI and the SI (AHI: ρ: −0.436; SI: ρ: −0.337, both *p* < 0.01). Among the participants with severe OSA (*n* = 21), a significant negative correlation was observed between the ArI and the overnight difference in acetone levels (ρ: −0.58, *p* < 0.01; Supplementary Figure S1).

### Associations between sleep quality indices and the overnight difference in breath acetone levels

Associations between the overnight difference in acetone levels and sleep quality indices were analyzed using a multivariable linear regression, adjusted for confounding factors (Table 4). A higher overnight difference in acetone levels was significantly associated with lower values of sleep quality indices. In Model 1, after adjustment for age, sex, BMI, neck circumference, and waist circumference, each one-unit increase in the overnight difference in breath acetone levels was significantly associated with a 12.69-event/h decrease in AHI values (95% CI: −24.92 to − 0.45, *p* < 0.05) and an 11.95-event/h decrease in ArI values (95% CI: −23.29 to − 0.62, *p* < 0.05). After additional adjustment for Type 2 diabetes (Model 2), the associations remained significant for the AHI and ArI (both *p* < 0.05). In Model 3, with further adjustment for alcohol consumption, each one-unit increase in overnight difference was associated with an 11.88-event/h decrease in the ArI values (95% CI: −23.23 to − 0.53) and a 12.63 decrease in AHI values (95% CI: −25.02 to − 0.23, both *p* < 0.05).

**Table 4:** Associations between sleep quality index values and the overnight difference in acetone levels (*N* = 66)

Variable	Model 1	Model 2	Model 3
Sleep quality index (events/h)			
AHI	−12.69 (−24.92 to −0.45)^*^	−12.66 (−24.95 to −0.37)^*^	−12.63 (−25.02 to −0.23)^*^
ODI	−2.29 (−18.63 to 14.06)	−2.21 (−18.28 to 13.86)	−2.62 (−17.94 to 12.69)
SI	−1.35 (−143.88 to 141.19)	−1.60 (−144.90 to 141.71)	−0.50 (−144.43 to 143.44)
ArI	−11.95 (−23.29 to −0.62)^*^	−11.99 (−23.31 to −0.66)^*^	−11.88 (−23.23 to −0.53)^*^

Data are expressed as beta coefficient (95% confidence interval)

*Abbreviations*: *AHI* Apnea-hypopnea index, *ODI* Oxygen desaturation index for ≥ 3%, *SI* Snoring index, *ArI* Arousal index

Model 1: Adjusted for age, sex, body mass index, neck circumference, and waist circumference

Model 2: Adjusted for age, sex, body mass index, neck circumference, waist circumference, and Type 2 diabetes

Model 3: Adjusted for age, sex, body mass index, neck circumference, waist circumference, Type 2 diabetes, and alcohol consumption

^*^p < 0.05

## Discussion

To further investigate metabolic associations between breath acetone and OSA manifestations, this study enrolled 66 participants with suspected OSA and collected their baseline characteristics and sleep parameters. Breath acetone levels were measured before and after sleep to assess their variations in relation to OSA manifestations. Significant differences were observed in demographic variables between the normal-to-mild and moderate-to-severe groups. Similarly, sleep parameters, particularly sleep quality indices, were significantly higher in the moderate-to-severe group than in the normal-to-mild group. The overnight difference in acetone levels was negatively associated with both AHI and ArI values, and these associations remained significant after adjustment for confounding factors.

The moderate-to-severe group consisted of more-elderly males and exhibited a higher mean BMI (27.63 ± 4.82 kg/m^2^), neck circumference (37.75 ± 3.96 cm), and waist circumference (93.94 ± 11.83 cm) than the normal-to-mild group. Regarding hypoxia- and arousal-related parameters, the moderate-to-severe group exhibited lower mean and minimum SpO_2_ levels, prolonged WASO (56.52 ± 30.28 min), and a higher proportion of wake stage during sleep (17.20% ± 8.67%). Similarly, the moderate-to-severe group had a significant higher AHI (36.96 ± 16.96 events/h), ODI (25.06 ± 15.82 events/h), and ArI (35.14 ± 16.72 events/h) compared to the normal-to-mild group. Previous studies reported significant correlations of sleep quality indices with age, BMI, neck circumference, and waist circumference [38, 39]. Age-related structural changes and fat distributions in the upper and central body regions are considered key factors driving these relationships [40, 41]. Altogether, aging and obesity were linked to OSA severity. Because multiple factors may influence breath acetone levels, individuals who had received a diagnosis of Type 1 diabetes or alcoholism were excluded from this study. Information regarding Type 2 diabetes status and alcohol consumption was obtained for reference. The prevalence of these conditions showed no significant differences between the two groups.

Regarding breath acetone levels, the overnight difference was significantly lower in the moderate-to-severe group. This difference was correlated with both ArI and AHI values, with a similar trend observed between the difference and AHI values in severe OSA patients. Furthermore, the results of a subgroup analysis among the participants without diabetes were consistent with those of the overall cohort. These findings suggest that Type 2 diabetes did not meaningfully affect breath acetone measurements or OSA manifestations in this study. In the regression models, negative associations were observed between the overnight difference in breath acetone levels and the AHI and ArI values after adjustment for baseline characteristics. These negative associations persisted after adjustment for Type 2 diabetes and alcohol consumption. Given that breath acetone can reflect ketone body metabolism [42], these findings suggest that lower ketone body availability during sleep was related to both high AHI and ArI values. Although the underlying mechanisms remain unclear, several potential reasons may partially account for these results. First, individuals with a higher BMI tend to exhibit lower breath acetone levels [43], suggesting that excess body weight may impair ketone production. Consistently, other studies reported lower breath acetone concentrations and delayed onset of ketosis in overweight individuals compared to those with a normal weight [44, 45]. Next, sleep fragmentation is associated with elevated nocturnal metabolism [46]. A related study observed a positive association between the AHI and resting energy expenditure, independent of factors including the BMI, age, and sex [47]. Similarly, OSA patients exhibited a significantly greater sleep energy expenditure compared to healthy controls [48]. Expanding on these findings, the high BMI and AHI observed in the moderate-to-severe group may contribute to decreased ketone body production and increased sleep energy expenditure, ultimately leading to lower breath acetone levels upon waking. Additionally, frequent arousals induced by respiratory disturbances and hypoxia in OSA patients activate the sympathetic nervous system [49, 50], potentially increasing energy demands [51]. Given the role of ketone bodies in modulating neuronal excitability [52], the reduced overnight difference in breath acetone levels may represent a compensatory metabolic response.

The present study has several strengths. First, to the best of our knowledge, this is the first study to investigate relationships between breath acetone levels and OSA manifestations derived from PSG. Furthermore, pre- and post-sleep breath acetone concentrations were measured using a calibrated portable device. By contrast, earlier studies primarily focused on differences in breath acetone levels between healthy individuals and those with OSA and on changes in breath acetone levels before and after sleep [53]. The major findings of this study are the negative associations between the overnight difference in acetone levels and both AHI and ArI values. These associations were also observed in the subgroup of participants without diabetes, indicating that hypoxia and arousal events may be linked to reduced breath acetone levels following sleep in this cohort.

This study has some limitations that should be addressed. First, although breath acetone measurements were supervised and followed a standardized protocol, variations in breathing maneuver (i.e., exhalation speed and volume) were not fully controllable, potentially introducing bias [54, 55]. Thus, future studies should incorporate repeated measurements to minimize these effects. Next, all participants were recruited from a single sleep center in northern Taiwan and had a similar ethnic background. In addition, sleep parameters were derived from a single-night PSG test. Although scoring was conducted by two well-trained technicians following the AASM manual, the first-night effect may have introduced bias, potentially influencing the accuracy of the results despite the moderate sample size (*n* = 66). Sleeping in an unfamiliar environment may induce anxiety, altering sleep architecture and arousal frequency, a phenomenon known as the first-night effect [56]. To enhance generalizability and mitigate this effect, future studies should involve larger, more-diverse populations and conduct multinight PSG assessments. Individuals with Type 1 diabetes or alcoholism were excluded from the current research, and all participants refrained from food and alcohol consumption prior to PSG. However, the study did not assess habitual dietary patterns, which may have affected the reliability and interpretation of the findings. Future research incorporating dietary records may enable a more comprehensive exploration of the associations between breath acetone levels and OSA manifestations.

## Conclusions

This study demonstrated that participants with moderate-to-severe OSA exhibited lower post-sleep breath acetone concentrations. Additionally, the overnight difference in acetone levels was negatively correlated with the AHI, SI, and ArI. In regression models, this overnight acetone concentration difference remained significantly associated with both AHI and ArI values after adjustment for cofounding factors. These findings shed light on potential relationships between breath acetone dynamics and OSA-related hypoxia and arousal events. However, further research is required to elucidate the underlying mechanisms and determine causal links between breath acetone alterations and OSA manifestations.

## Supplementary Information

Supplementary Material 1.

## Data Availability

All data were collected at the Sleep Center of Taipei Medical University–Shuang Ho Hospital between May 2024 and January 2025. Due to the inclusion of personal information, the dataset generated and/or analysed during the current study is not publicly available. Interested researchers can contact the corresponding author to request access to the dataset or related documents.

## Abbreviations

AHI: Apnea-hypopnea index
ArI: Arousal index
BMI: Body-mass index
CI: Confidence interval
ODI: Oxygen desaturation index for ≥ 3%
OSA: Obstructive sleep apnea
PSG: Polysomnography
REM: Rapid-eye-movement
SI: Snoring index
SpO_2_: Oxygen saturation measured by pulse oximetry
WASO: Waking after sleep onset
WO_3_: Tungsten trioxide
