# Supplementary material for: Associations between breath acetone overnight dynamics and obstructive sleep apnea manifestations
Source: BMC Pulm Med. 2025 Nov 27;25:548. doi: 10.1186/s12890-025-04021-0 (PMC12659279; doi:10.1186/s12890-025-04021-0)

**Supplementary Materials**

**Table S1. Breath Acetone Levels and Sleep Quality Index Values Among Participants Without Diabetes, Stratified by Obstructive Sleep Apnea Severity (*N* = 58)**

| Variable | Normal-to-mild group  (*N* = 21) | Moderate-to-severe group  (*N* = 37) | *p* value |
| --- | --- | --- | --- |
| **Acetone level (ppm)** | | | |
| Presleep | 2.29 ± 0.31 | 2.36 ± 0.46 | 0.99 |
| Postsleep | 2.32 ± 0.43 | 2.13 ± 0.37 | 0.09 |
| Overnight Difference | 0.03 ± 0.26 | −0.23 ± 0.28 | <0.01 |
| **Sleep quality index (events/h)** | | | |
| AHI | 7.06 ± 4.24 | 37.21 ± 17.51 | <0.01 |
| ODI | 11.56 ± 26.39 | 25.82 ± 16.20 | <0.01 |
| SI | 59.31 ± 126.43 | 255.40 ± 186.95 | <0.01 |
| ArI | 24.10 ± 13.41 | 34.98 ± 17.75 | 0.02 |
| Abbreviations: AHI, apnea–hypopnea index. ODI, oxygen desaturation index ≥ 3%. SI, snoring index. ArI, arousal index.  Data are presented as the mean ± standard deviation or number (percentage).  *p* values are derived from the Mann–Whitney U-test. | | | |

**Table S2. Correlations Between Sleep Quality Index Values and Breath Acetone Levels Among Participants Without Diabetes (*N* = 58)**

| Variable | **Acetone level (ppm)** | | |
| --- | --- | --- | --- |
|  | Presleep | Postsleep | Overnight difference |
| **Sleep quality index (events/h)** | | | |
| AHI | −0.002 | −0.24 | −0.436 ^**^ |
| ODI | −0.104 | −0.194 | −0.187 |
| SI | −0.114 | −0.259 | −0.337 ^**^ |
| ArI | 0.194 | −0.02 | −0.438 ^**^ |
| Abbreviations: AHI, apnea–hypopnea index. ODI, oxygen desaturation index ≥ 3%. SI, snoring index. ArI, arousal index.  Data are expressed as coefficients.  ^**^ *p* < 0.01. | | | |

**Figure S1: Correlation Between Arousal Index (ArI) Values and Overnight Differences in Breath Acetone Levels Among** **Participants With Severe Obstructive Sleep Apnea (OSA) Without Diabetes**

Among the participants with severe OSA (apnea–hypopnea index (AHI) ≥ 30 events/h, *n* = 21) without diabetes, ArI values were significantly and negatively correlated with overnight changes in breath acetone levels.


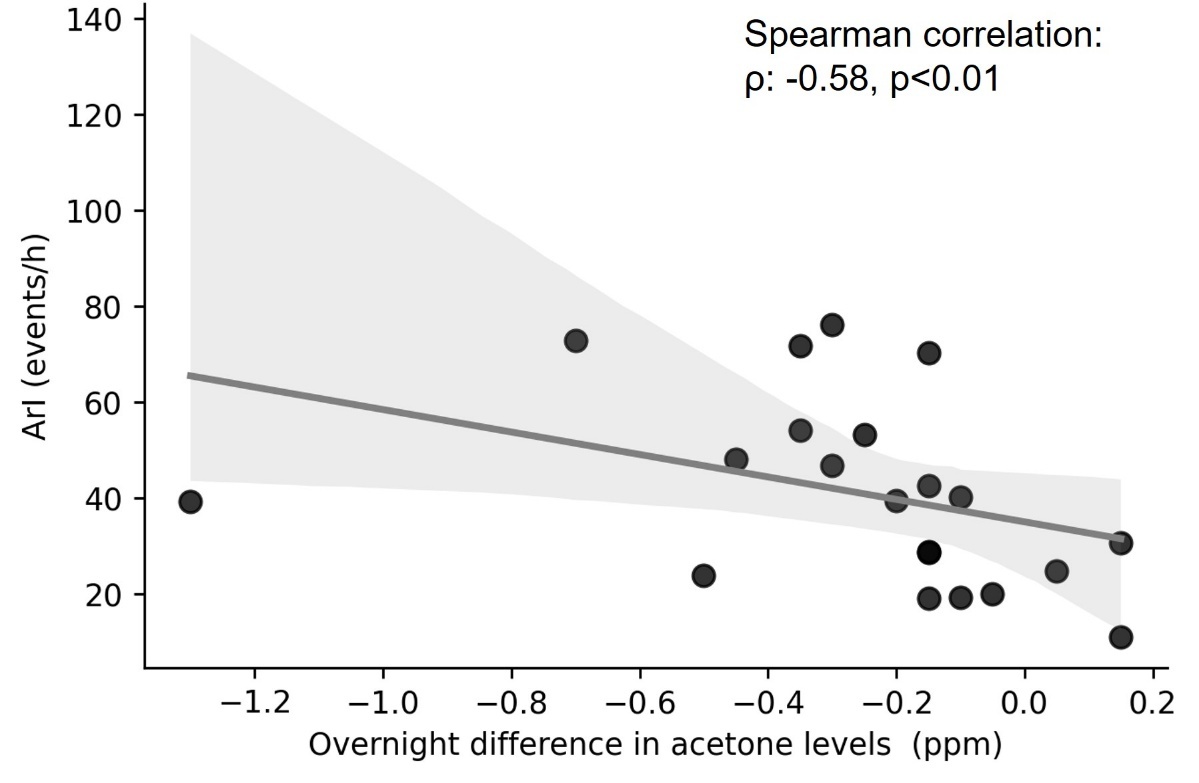

Supplement: Supplementary file 1 — Supplementary Material 1. [file 12890_2025_4021_MOESM1_ESM.docx]
